# Proteomic analysis of ovarian carcinoma reveals diagnostic and prognostic biomarkers with histotype- and stage-specificity

**DOI:** 10.1186/s13048-026-01984-4

**Published:** 2026-01-28

**Authors:** Lucas Werner, Ella Ittner, Hugo Swenson, Elisabeth Werner Rönnerman, Claudia Mateoiu, Anikó Kovács, Pernilla Dahm-Kähler, Per Karlsson, Toshima Z. Parris, Khalil Helou

**Affiliations:** 1 Department of Oncology, Institute of Clinical Sciences, Sahlgrenska Academy, University of Gothenburg, Gothenburg, Sweden; 2 Sahlgrenska Center for Cancer Research, Sahlgrenska Academy, University of Gothenburg, Gothenburg, Sweden; 3 Department of Clinical Pathology, Sahlgrenska University Hospital, Region Västra Götaland, Gothenburg, Sweden; 4 Institute of Biomedicine, Sahlgrenska Academy, University of Gothenburg, Gothenburg, Sweden; 5 Department of Obstetrics and Gynecology, Institute of Clinical Sciences, Sahlgrenska Academy, University of Gothenburg, Gothenburg, Sweden; 6 Department of Oncology, Sahlgrenska University Hospital, Region Västra Götaland, Gothenburg, Sweden

## Abstract

**Background:**

Epithelial ovarian cancer (EOC) is the deadliest gynecologic cancer, due to asymptomatic early stages, vague symptoms in later stages, and limited clinical tools. Despite distinct clinicopathologic features, all EOC histotypes typically receive identical primary treatment, and are often studied as a single entity.

**Methods:**

We analyzed the proteome of 244 patients and identified differentially abundant proteins (DAPs) with and without stage specificity across histotypes and constructed panels of DAPs to distinguish histotypes in both early and late stages. Survival analysis was performed to find proteins associated with clinical outcomes, and enrichment analysis was conducted to reveal biological processes connected to prognosis and the proteins involved.

**Results:**

Here we find DAPs without (e.g. S100A1, AGR2, CTH) and with (TSPYL, VWA2, GPC6, S100P) stage-specificity for each histotype. Survival analysis revealed histotype- and stage-specific prognostic markers (e.g., EXO3CL2, PPIL6, GYG1, GAPDH), while biological process enrichment highlighted pathways underlying clinical outcomes.

**Conclusions:**

Our findings provide novel diagnostic and prognostic biomarker candidates and insights into mechanisms driving EOC progression with histotype- and stage-specificity. This may aid the development of improved clinical tools for detection, patient stratification, and targeted therapies in EOC.

**Supplementary Information:**

The online version contains supplementary material available at 10.1186/s13048-026-01984-4.

## Introduction

Epithelial ovarian cancer (EOC) is the most lethal gynecologic malignancy worldwide [1]. Its relatively low 5-year survival rate (45%) is partly attributed to the lack of sensitive diagnostic tools, with only CA-125 and HE4 being used clinically, both of which lack specificity and are not consistently detected in early stages [2, 3]. EOC is asymptomatic in early stages (I/II), and presents vague symptoms at late stages (III/IV) such as abdominal pain and bloating that are often mistaken for less severe conditions [4]. Despite efforts to increase the sensitivity and specificity of CA-125 and the introduction of HE4 as a complementary biomarker, approximately 70% of patients are still diagnosed at an advanced stage [5]. In 2020, the World Health Organization (WHO) established updated guidelines for the subclassification of EOC into five biologically and clinical distinct histotypes: high-grade serous carcinoma (HGSC), low-grade serous carcinoma (LGSC), endometrioid carcinoma (EC), mucinous carcinoma (MC), and clear-cell carcinoma (CCC) [6]. While this classification has improved our understanding of EOC heterogeneity, it has yet to significantly influence treatment strategies. Still, primary treatment for EOC patients, which is commonly cytoreductive surgery followed by platinum-based chemotherapy, is similar regardless of histotype [7]. Importantly, complete cytoreduction is a significant prognostic factor in EOC [8]. To date, only targeted therapy with Poly ADP-ribose polymerase (PARP) inhibitors has been introduced as a new therapeutic option, showing improved progression-free survival for women with germline BRCA1/2 mutations, a trait mainly linked to HGSC [9, 10].

Early-stage EOC is most commonly classified as EC (stage I: 40%; stage II: 40%), MC (I: 80%; II: 10%), and CCC (I: 70%; II: 20%), while the most common histotype, HGSC, is overrepresented in stage III (70%), thereby leading to poor prognosis for HGSC patients compared to the other histotypes [3, 10]. EC, MC, and CCC are not only associated with better prognosis due to their early detection, but also for the increased chemosensitivity of EC. However, advanced-stage CCC is typically resistant to platinum-based chemotherapy and has a prognosis comparable to HGSC and EC at advanced stage [5, 11]. Consequently, favorable 10-year survival rates have been observed for early-stage patients with MC (> 90%) and CCC (80–90%), intermediate survival for EC (60–70%), and poor 5-year survival for HGSC (40%). At late stages, 10-year survival is extremely poor for MC (< 20%) and CCC (10%)[10], further highlighting the importance of considering both histotype and stage during patient management. Nevertheless, little research has been devoted to developing clinical tools tailored to each histotype and disease stage, with most studies focusing on HGSC [12, 13].

Efforts have been made to identify novel biomarkers by profiling the EOC proteome. Studies to measure protein abundances in plasma samples have led to the development of biomarker panels that outperform CA-125 in detection sensitivity but still lack histotype-specificity [14, 15]. In recent years, large-scale proteomic studies have been conducted across all EOC histotypes, stages, and tissue types, resulting in proposed biomarker panels and prognostic biomarkers that have been validated in serum. However, these studies have not prioritized the development of histotype- and stage-specific candidates [16, 17].

In this study, we employed four-dimensional data-independent acquisition (4D-DIA) proteomics to evaluate protein abundances in 244 primary EOC samples, stratified by histotype (HGSC, EC, MC, and CCC) and stage (I–IV). We identified novel deregulated proteins specific to each histotype at both early and late stages. Survival analysis revealed stage-specific prognostic proteins associated with favorable or unfavorable outcomes for each histotype. Gene Ontology (GO) enrichment analysis uncovered biological processes underlying prognosis and the involvement of these proteins. Together, these findings highlight putative biomarkers with potential to serve as histotype- and stage-specific clinical tools to improve diagnosis, guide treatment decisions, and identify therapeutic targets and mechanisms driving disease progression. An overview of the experimental and analytical workflow can be found in Fig. S1.

## Methods

### Patient cohort

Fresh-frozen primary tumor tissues from 244 patients diagnosed with ovarian carcinomas between 1993 and 2022 were collected from the tumor bank at the Sahlgrenska University Hospital Oncology lab (Gothenburg, Sweden). Clinicopathologic data were obtained from the Swedish Quality Register for Gynecological Cancer National Quality Registry at the Regional Cancer Center West (Gothenburg, Sweden) and the Cancer Registry at the National Board of Health and Welfare. Stage upon diagnosis was determined using the International Federation of Gynecology and Obstetrics (FIGO) guidelines. Overall survival times were defined as number of days after diagnosis until the date of death by any cause, whereas disease-specific survival used death caused by ovarian carcinoma.

All tumor specimens were reclassified by a board-certified pathologist at Sahlgrenska University Hospital according to the 2020 World Health Organization (WHO) guidelines using 4 µm full-faced formalin-fixed paraffin-embedded (FFPE) tissue paired to the fresh-frozen samples. If no FFPE was available for a patient, hematoxylin and eosin staining of fresh-frozen tumor tissue was subjected to formalin fixation and paraffin embedding.

### Sample preparations and running bottom-up liquid chromatography and mass spectrometry

Tumor tissue pieces of approximately 2 × 2x2 mm were first homogenized using a Covaris ML230 ultrasonicator with tissue in 2% sodium dodecyl sulfate and 50 mM triethylammonium bicarbonate (TEAB). To determine protein concentration of the resulting lysates, bicinchronic acid (BCA) protein assay was utilized using the Pierce BCA Protein Assay Kit (ThermoFisher Scientific). Lysate volumes containing 50 µg of protein for each sample were subsequently used for reduction and alkylation before enzymatic digestion. Proteins were reduced in 10 mM dithiothreitol at 56 °C for 30 min and then alkylated in 20 mM chloroacetamide at room temperature for 10 min. Protein samples were added to washed hydrophobic and hydrophilic Sera-Mag SpeedBeads (Carboxylate-Modified, Cytiva) in a 10:1 bead-to-protein ratio. The clean-up employed the SP3 workflow tailored to mass spectrometry [18] where proteins were precipitated on the beads by ethanol and washed and left to dry at room temperature. Digestion of proteins to peptides on the beads was done with 1 µg of LysC + Trypsin (Promega and ThermoFisher Scientific, respectively) in 50 mM HEPES + 1 M Urea by incubating at 37 °C overnight while shaking. Then, 1 µg of Trypsin was added to incubate for an additional three hours. Concentration of purified and eluted peptides were determined with a Pierce Quantitative Peptide Assay (ThermoFisher Scientific).

Liquid chromatography-mass spectrometry (LC–MS) was performed on an Evosep One liquid chromatography (LC) system (Evosep) coupled to a timsTOF HT mass spectrometer (Bruker). The LC system was equipped with a Pepsep C18 column (15 cm × 150 um ID, 1.5 µm particle size). In total, 400 ng peptides was dissolved in 0.1% formic acid (FA) and 0.15% n-Dodecyl-beta-Maltoside (DDM) and was loaded onto Evotips Pure (Evosep) according to the manufacturers’ instructions. The LC system ran the 30 samples per day (30SPD) method. The timsTOF was run in DIA-PASEF mode with 10 PASEF/MSMS scans. The samples were matched using directDIA (Swissprot, June 2023, 20,407 entries) with Spectronaut (v. 18.6.231227.55695). Strict trypsin with 1 missed cleavage for protein digestion was set. Variable modifications were set to Methionine oxidation and N-terminus acetylation, and carbamidomethylation of cysteine for fixed modification. The retention time and ion-mobility value were automatically selected and only b- and y-ions were used. Default settings were used for identification and matching towards directDIA spectra libraries and quantification was performed on Only Protein Group Specific.

### Data pre-processing and filtering

The data were normalized using cross-run normalization with no filter type, which is based on local regression performed during runs in directDIA mode in Spectronaut (v. 18.6.231227.55695). The intensity distribution across the sample cohort after normalization yielded consistent average intensities across all samples, as shown in Fig. S2. After running the protein identification, intensities from MS2 were obtained along with the protein annotations PG.ProteinGroups, PG.Genes, and PG.ProteinDescriptions for further pre-processing and analyses. Using the R package Prostar (v. 1.34.6), the data were filtered to retain only the proteins having a detected abundance in at least 70% of the samples for at least one group (here histotype). Filtered data were then log2-transformed. All subsequent analyses were conducted using R (v. 4.3.3).

### Differential protein abundance analysis

After inspecting data distribution, differential abundance (DA) analysis was done for histotypes categorized into early-stage (I or II) and late-stage (III or IV) samples. Unpaired limma t-tests were performed for the full proteome using NormalyzerDE (v. 1.20.0), generating fold change (FC) and two-sided empirical Bayes moderated t-test p-values for all identified proteins after filtering that were then subjected to false discovery rate (FDR) correction. For each histotype, the abundance of a protein for early-stage patients was compared to the abundance of early-stage samples for the other histotypes combined. This process was repeated for late-stage samples to identify differentially abundant proteins (DAPs) that are stage-specific. Additionally, pairwise comparisons between all histotypes within the two stage categories was done, as well as comparing early- and late-stage samples within each histotype. Significance thresholds were FDR < 0.05, and FC > 1.5 for upregulated and FC < −1.5 for downregulated proteins. Custom heatmaps were produced using ComplexHeatmap (v. 2.18.0) and boxplots with ggplot2 (v. 3.5.1), where pairwise comparisons between the log2 intensities of the proteins between histotypes were made with the Wilcoxon test and p-values were adjusted for multiple testing using FDR for the boxplots.

### Identifying biomarker panels for all comparisons

DAPs from the DA analysis were used to acquire panels of proteins that can be used to distinguish a histotype from the others or in pairwise comparisons in both early- and late-stage disease. A custom iterative support vector machine (SVM) modeling approach was used. Using all identified DAPs for a histotype in either early- or late stage, logistic regression with least absolute shrinkage and selection operator (LASSO) using elastic net (alpha = 0.5) was used to pre-select DAPs using glmnet (v. 4.1–8). The logistic regression applied fivefold cross-validation and area under the curve (AUC) for classification and lambda min to select proteins with non-zero coefficients. These proteins were then used to train SVM models using kernlab (v. 0.9–33) to identify the combination of proteins with the highest AUC score. The models were trained on abundance data comprising 80% of the dataset, and tested on the remaining 20% to evaluate performance with confusion matrices generated by caret (v. 6.0–94) for sensitivity and specificity, and pROC (v. 1.18.5) for AUC scores and plotting receiver operating characteristics (ROC) curves. The models first identified the protein generating the highest AUC scores independently from the others. Then, the other pre-selected proteins from LASSO were iteratively added to the model one at a time and the AUC score recalculated. If the AUC score improved the protein, it was kept in the model. This was repeated until there were no more proteins that improved the model. In a second step, additional proteins were added to the SVM model as long as the AUC score did not decrease more than one percent unit below the maximum achieved score. This was done to increase the panel sizes without compromising predictive power. A cap of a maximum of 5 proteins in the model was used to reflect panels that would be more feasible in a clinical setting. These selected biomarkers were used in a similar SVM modeling pipeline with kernlab (v. 0.9–33) to simulate the theoretical number of samples required to achieve a high and stable AUC score (≥ 0.95). Here a smaller training set (70%) was used, utilizing the log2 abundances of the selected biomarkers for each histotype. Average AUC scores were estimated by repeating the procedure 200 times using different sample sizes (5, 10, 15, 20, 25, 30, 40, 50, and 60 samples), with standard deviations also derived.

### Survival analysis and validation of model robustness

Multivariate Cox regression was performed to obtaining histotype- and stage-specific proteins associated with overall- and disease-specific survival. For each histotype in early- and late stage separately, Cox proportional hazard models were constructed with coxph (v. 3.7–0) for the full proteome, where the survival models were based on the protein abundance and adjusted for the covariates patient age and tumor stage. The administrative cut-off date for follow-up was 2024–03–24. This generated an association with survival based on Wald test p-value along with the estimated hazard ratio (HR), confidence interval (CI) for HR, FDR, and concordance index (C-index) for all proteins by first selecting the proteins and covariates with non-zero coefficients using least absolute shrinkage and selection operator (LASSO). Additionally, the robustness of the survival models was assessed by running the Cox models on bootstrapped samples in 1000 iterations and the average p-value from all models were used to estimate a bootstrap p-value. A protein was deemed significantly associated with survival when presenting an FDR < 0.05, bootstrap p-value < 0.2, and HR < 1 for decreased risk and HR > 1 for increased risk of death (CI not spanning 1). Forest plots were generated with forestplot (v. 3.1.6). Kaplan–Meier curves were plotted using ggplot2 (v. 3.5.1), with log2 abundances dichotomized into high and low using the median log2 abundance for the histotype for the stage-group presented.

### Gene ontology enrichment analysis for proteins significantly associated with survival

Proteins deemed significantly associated with either decreased or increased risk of death were used to perform gene ontology (GO) enrichment analysis for each histotype in early- and late stage. Enrichment analysis was performed with the proteins for the given histotype and stage group using the enrichGO-function in clusterProfiler (v. 4.10.1), obtaining GO annotations with org.Hs.eg.db (v. 3.18.0). Enrichment was done on biological processes (BPs), and an FDR cutoff of 0.05 was set as significance threshold. The five GO terms of highest significance for respective histotype in each stage group in overall- and disease-specific survival were used to make dotplots using ggplot2 (v. 3.5.1) with imported descriptions from the GO.db package (v. 3.18.0). The proteins involved with each pathway were used to identify DAPs involved in the enriched biological processes.

### Statistics and reproducibility

The statistical analyses and modeling were performed as described in the subsections above. For a more detailed description, please see the publicly available code provided.

## Results

### Patient cohort

The cohort was comprised of 244 patients diagnosed with the four main EOC histotypes, i.e., HGSC (*n* = 122), EC (*n* = 42), MC (*n* = 35), and CCC (*n* = 45) at both early stage (stage I or II; *n* = 92) and late stage (stage III or IV; *n* = 152). Although LGSC was originally included in the cohort design, too few samples were available for robust statistical analysis and it was therefore excluded from this study. The mean age of the patient cohort was between 59 and 61 years, with more ECs (50%) diagnosed at an early stage than the other histotypes (range, 29–39%). A detailed overview of the clinical characteristics of the cohort are shown in Data S1.

### Differential abundance analysis

**Fig. 1:**
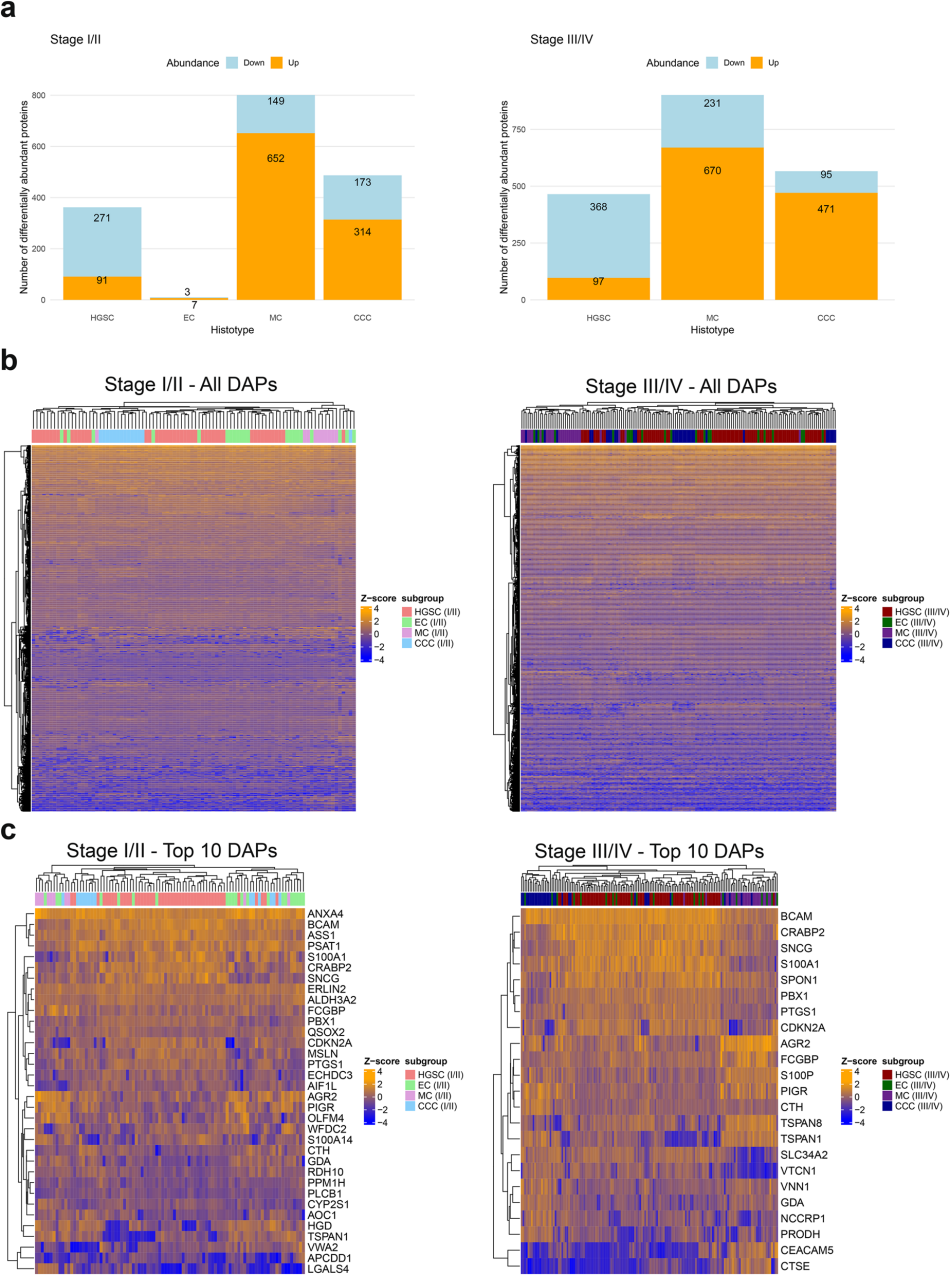
Differential abundance analysis for epithelial ovarian carcinoma histotypes at early- (I/II) and late-stage (III/IV) disease. **a** The number of identified DAPs for each histotype when comparing early- and late-stage samples. Differential abundance in each stage group was estimated by comparing only samples belonging to the given stage group for each histotype. Heatmaps showcasing the clustering of samples for histotypes based on (**b**) the abundance of identified DAPs and (**c**) the ten most differentially abundant proteins (five most upregulated and five most downregulated) based on log2 FC at early-stage (left) and late-stage (right). CCC Clear cell ovarian carcinoma, DAP Differentially abundant protein, EC Endometrioid ovarian carcinoma, FC Fold change, HGSC High-grade serous ovarian carcinoma, MC Mucinous ovarian carcinoma

Mass spectrometry analysis and protein identification yielded 305,873 unique peptides mapping to 12,707 protein IDs, with 10,308 proteins remaining after filtering for those displaying quantities in at least 70% of the samples in at least one histotype (Data S2). Projections of principal component analysis (PCA) and unform manifold approximation and projection (UMAP) suggest mostly uniform abundance patterns within each histotype, particularly in the non-linear UMAP representation (Fig. S3). This underlines low risk of batch effects or systematic biases. Differential abundance analysis revealed significantly deregulated DAPs (differentially abundant proteins; FDR < 0.05, FC >|1.5|) for all four histotypes across both stage groups, except for late-stage EC. Late-stage MC had the highest number of DAPs (n = 901) and early-stage EC the fewest (n = 10; Fig. 1a). Hierarchical clustering of the DAPs showed overlapping abundances between HGSC and EC, and distinct clusters for MC and CCC in early stage. In late stage, only MC presented uniform grouping (Fig. 1b). Using the five most up- and downregulated proteins resulted in improved clustering of the histotypes, particularly for HGSC in early stage, but less distinct clusters for MC. For late-stage disease, the most differentially abundant proteins yielded clusters with the clearest separation of CCC. Protein abundance patterns for HGSC and EC were similar, resulting in overlapping clusters (Fig. 1c, Table S1).

S100A1, SNCG, and CRABP2 were three of the most differentially abundant proteins at both early- and late-stage disease (Fig. 1c), and had the highest log2 FC in HGSC (both stage groups). AGR2 and CTH were the most upregulated DAPs in MC and CCC (both early- and late stage), respectively. Additionally, AGR2 had the lowest abundance in HGSC across the stages. Inversely, S100A1 was the lowest abundant protein in MC (Table S1). At early stage, TSPYL5 and GSTA1 were the most differentially abundant and unique proteins (highest and lowest log2 FC) for HGSC, VWA2 and MSLN for EC, SCGB1D2 and SPON1 for MC, and PIGR and WFDC2 for CCC. At late stage, unique DAPs included IL4I1 and CTSE for HGSC, GPC6 and VTCN1 for MC, and S100P and CYS1 for CCC (Table S2; Data S3). Additionally, DAPs between early- and late stage for EC and MC were found when comparing the stage groups within each histotype. VWA2, which was also the most upregulated protein in EC when compared to the other histotypes at early stage, was also upregulated when compared to EC at late stage. In MC, WFDC2 was upregulated in early-stage and TRIQK in late-stage (Fig. S4; Data S4).

Differential abundance analysis for the pairwise comparisons of the histotypes was also performed. DAPs were found in all comparisons in both early- and late-stage disease. However, only two proteins were identified between HGSC and EC at a late stage, i.e., CRACR2B and TRIM2 (both upregulated in EC; Data S4). A summary of the DAPs of highest and lowest differential abundance for each comparison can be found in Table S1 and S2.

### Predictive power of histotype- and stage-specific biomarker panels

**Fig. 2:**
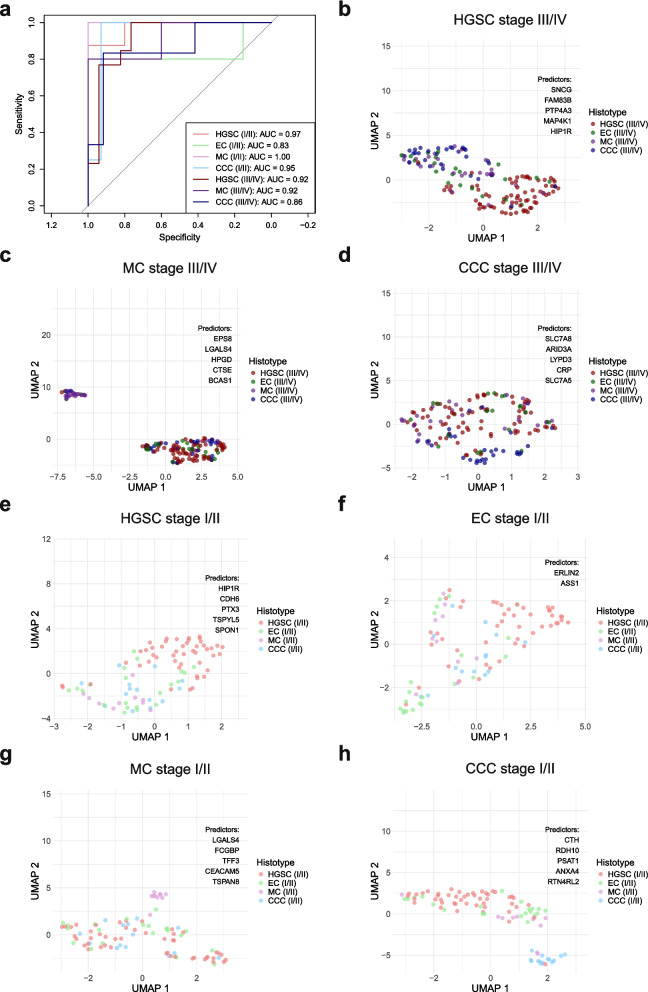
Identification of biomarker panels that stratify a histotype from the others at early- and late stage using support vector machine models. **a** ROC curves with AUC scores for each model based on the abundance of selected proteins for each histotype in early- and late-stage samples. UMAP for the abundance profiles at late-stage of the selected proteins for **b** HGSC, **c** MC, and **d** CCC biomarker panels. UMAPs for the biomarker panels for early-stage samples are displayed for **e** HGSC, **f** EC, **g** MC, and **h** CCC. AUC Area under the curve, CCC Clear cell ovarian carcinoma, EC Endometrioid ovarian carcinoma, HGSC High-grade serous ovarian carcinoma, MC Mucinous ovarian carcinoma, ROC Receiver operating characteristics, UMAP Uniform manifold approximation and projection

To define combinations of up- and downregulated DAPs that distinguish a histotype from the others, support vector machine (SVM) models were constructed that identify panels of DAPs with the highest area under the curve (AUC) score (> 0.70). With the exception of EC that only had DAPs at early stage, such panels were established for each histotype at both early- and late stage: HGSC (early stage: HIP1R, CDH6, PTX3, TSPYL5, SPON1; late stage: SNCG, FAM83B, PTP4A3, MAP4K1, HIP1R), MC (early stage: LGALS4, FCGBP, TFF3, CEACAM5, TSPAN8; late stage: EPS8, LGALS4, HPGD, CTSE, BCAS1), CCC (early stage: CTH, RDH10, PSAT1, ANXA4, RTN4RL2; late stage: SLC7A8, ARID3A, LYPD3, CRP, SLC7A5), and EC (early stage: ERLIN2, ASS1). In general, the performance based on AUC scores was poorer for models at late-stage, although the panel for EC at early-stage had the lowest AUC score (0.83). At early-stage, the panels for MC and CCC had near perfect predictive power (AUC = 1 and 0.95, respectively) unlike at late-stage (AUC = 0.92 and 0.86, respectively). It is important to note that the sample sizes for early-stage MC (n = 10) and CCC (n = 14) were small, and this is likely contributed to overestimation of AUC values. Predicted sample sizes required for high discriminative power exceeded the availabe numbers for these groups (Data S5, Fig. S5), indicating that findings for these panels should be interpreted with caution. For HGSC, the performance of the proteins for early- and late stage were comparable (Fig. 2a). Poor clustering of protein abundance patterns across samples for early-stage panels was observed for all histotypes when projected on a UMAP, except for MC which had clustering (Fig. 2b-d). At early-stage, the abundance of proteins selected for HGSC and EC were spread but still formed distinct clusters separate from the other histotypes (Fig. 2e, f). MC and CCC, on the other hand, presented distinct clusters (Fig. 2g, h; Data S5).

**Fig. 3:**
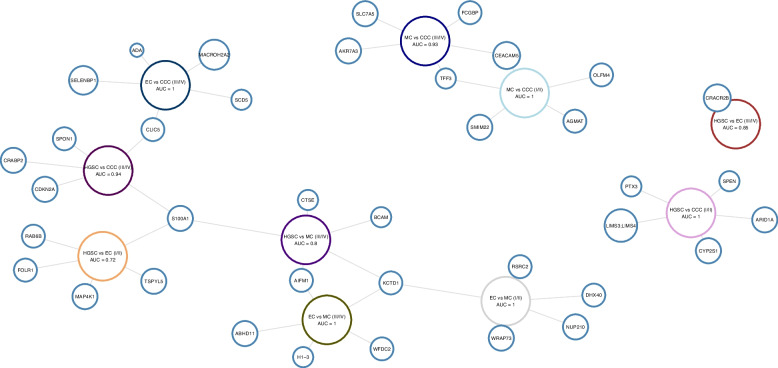
Network graph of the selected differentially abundant proteins for stratifying histotypes in pairwise comparisons at early- and late-stage, along with AUC scores for the support vector machine models generated by the proteins. The nodes indicate comparison origin as well as overlap with selected proteins for the models in other comparisons. AUC Area under the curve, CCC Clear cell ovarian carcinoma, EC Endometrioid ovarian carcinoma, HGSC High-grade serous ovarian carcinoma, MC Mucinous ovarian carcinoma

The same SVM modeling was applied for DAPs from pairwise comparisons, though the comparisons HGSC vs. MC and EC vs. CCC at early-stage were excluded due to small sample size. Here, great caution should be taken when interpreting panels for comparisons with early-stage MC and CCC, as the AUC scores are likely overestimated, as highlighted by the predicted number of required samples (Data S5, Fig. S6). The predictive power for the biomarker panels was high (AUC > 0.80) for both early- and late-stage comparisons, with only HGSC vs. EC at early stage showing poor performance with an AUC score of 0.72. Notably, S100A1 was selected for late-stage panels when HGSC was compared to MC and CCC as well as for HGSC vs. EC at early stage. TFF3 and CEACAM5 could stratify MC and CCC at both early- and late stage. Although KCTD1 appeared to be a key protein for distinguishing between EC and MC across all stages, it was also included in the SVM model for HGSC vs. MC at late stage. Of all the biomarker panels, the selected proteins for HGSC vs. CCC at early stage and HGSC vs. EC at late stage were completely unique for these comparisons (Fig. 3; Data S5).

### Identification of prognostic biomarker candidates for overall- and disease-specific survival

**Fig. 4:**
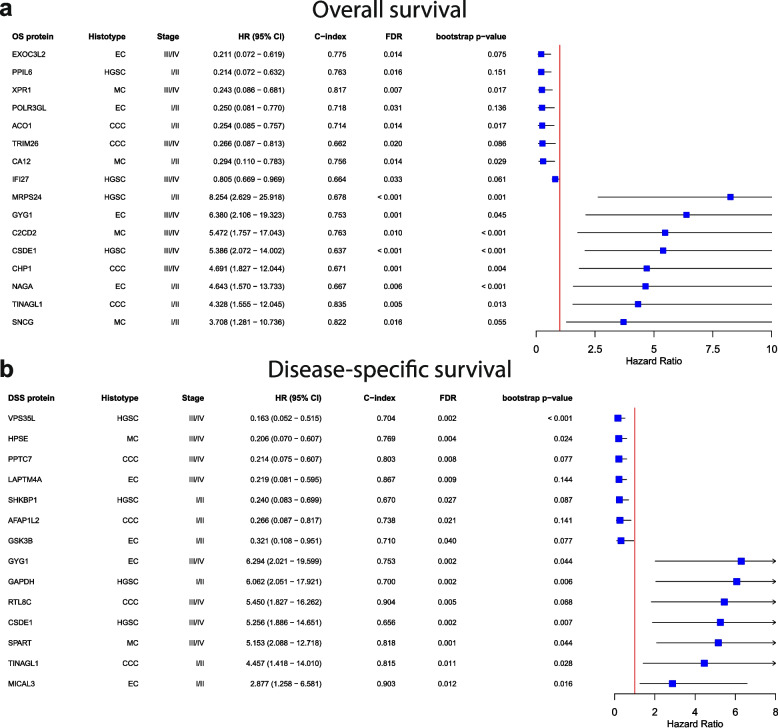
Forest plots for proteins associated with the highest increased risk (HR > 1) and decreased risk (HR < 1) of death for histotypes in early- and late-stage based on multivariate Cox regression. Survival metrics for the proteins of highest and lowest estimated HR for each histotype and stage group for (**a**) OS and (**b**) DSS, ranked from lowest to highest for decreased risk of death, and highest to lowest for increased risk of death. All survival models were based on the protein abundance (low and high) and adjusted for age and stage of the patients if selected by LASSO, and models were validated by bootstrapping with 1,000 iterations. FDR correction of p-values was done by including all proteins with a non-zero coefficient in LASSO when running Cox regression for the full proteome for each histotype separately. CCC Clear cell ovarian carcinoma, C-index Concordance index, CI Confidence interval, DSS Disease-specific survival, EC Endometrioid ovarian carcinoma, FDR False discovery rate, HGSC High-grade serous ovarian carcinoma, HR Hazard ratio, LASSO Least absolute shrinkage and selection operator, MC Mucinous ovarian carcinoma, OS Overall survival

To determine whether protein expression patterns affect clinical outcome (overall survival [OS] and disease-specific survival [DSS]) based on histotype and stage, survival analysis was performed using multivariate Cox regression adjusted for patient age and tumor stage (Data S6). At early-stage, proteins significantly associated (FDR < 0.05, bootstrap p-value < 0.20) with increased (HR > 1) or decreased (HR < 1) risk of death were found for all histotypes in both stage groups except MC in early stage for DSS (Table S3, Data S7). Of these, the proteins presenting the highest increased and decreased risk of death were identified. For OS, highest decreased risk of death was found for EXO3CL2 (EC) at late stage and PPIL6 (HGSC) at early stage, while the highest increased risk of death was found for GYG1 (EC) at late stage and MRPS24 (HGSC) at early stage. Notably, SNCG, an upregulated protein in HGSC for both stage groups, was associated with the highest increased risk of death in MC at early-stage (Fig. 4a). Counterparts for DSS included VPS35L (HGSC) at late-stage and SHKBP1 (HGSC) at early-stage for decreased risk, and GYG1 (EC) at late-stage and GAPDH (HGSC) at early-stage (Fig. 4b). In both OS and DSS, significant survival-associated proteins showed moderate model stability across all histotypes, with most C-index values ranging between 0.70–0.80. However, for HGSC the predictive performance was lower in both stage groups, with C-index ≤ 0.7 for most proteins (Data S7).

**Fig. 5:**
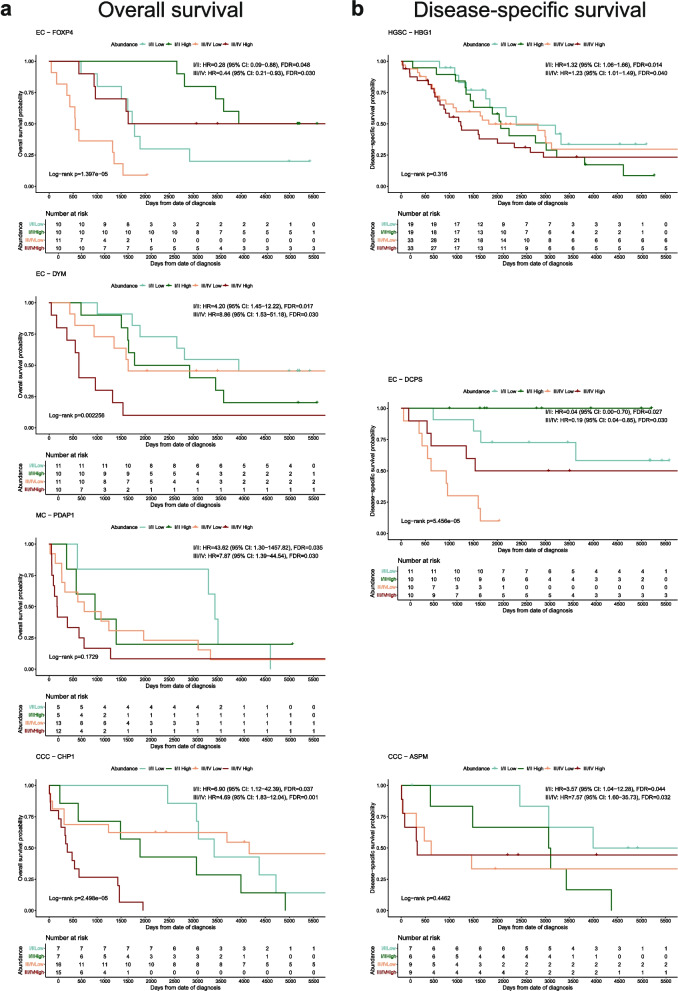
Kaplan Meier plots of proteins significantly associated with increased or decreased risk of death (HR >|1|, FDR < 0.05, bootstrap p-value < 0.20) for early- and late-stage patients that had a positive correlation of estimated HR for the stage groups. Proteins where HR at both early- and late-stage either indicating increased or decreased risk of death for (**a**) OS and (**b**) DSS. Displayed HR, CI, and FDR were estimated from multivariate Cox regression, with Cox proportional hazard models adjusted for age and stage. Log-rank p-values are global p-values for the four abundance groups. CCC Clear cell ovarian carcinoma, CI Confidence interval, DSS Disease-specific survival, EC Endometrioid ovarian carcinoma, FDR False discovery rate, HGSC High-grade serous ovarian carcinoma, HR Hazard ratio, MC Mucinous ovarian carcinoma, OS Overall survival

**Fig. 6:**
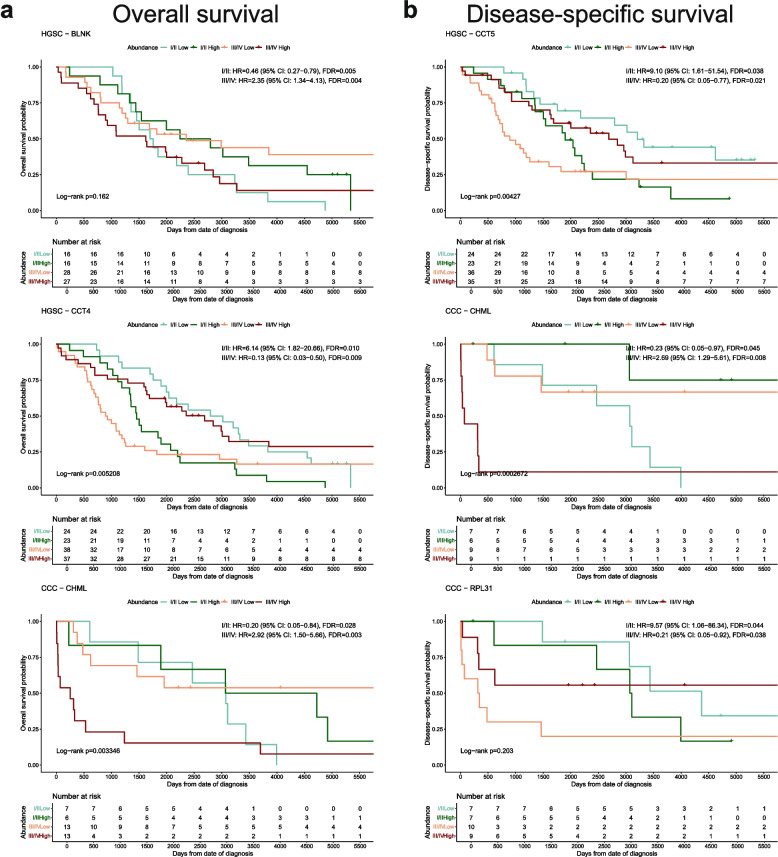
Kaplan Meier plots of proteins significantly associated with increased or decreased risk of death (HR >|1|, FDR < 0.05, bootstrap p-value < 0.20) for early- and late-stage patients that have a negative correlation of estimated HR for the stage groups. Proteins where HR in early- and late-stage had opposite directionality in (**a**) OS and (**b**) DSS. Displayed HR, CI, and FDR were estimated from multivariate Cox regression, with Cox proportional hazard models adjusted for age and stage. Log-rank p-values are global p-values for the four abundance groups. CCC Clear cell ovarian carcinoma, CI Confidence interval, DSS Disease-specific survival, EC Endometrioid ovarian carcinoma, FDR False discovery rate, HGSC High-grade serous ovarian carcinoma, HR Hazard ratio, MC Mucinous ovarian carcinoma, OS Overall survival

Proteins with a positive correlation (either HR > 1 or HR < 1 in both stage groups) in increased or decreased risk of death were identified in early- and late stage for some histotypes. Such correlation for HR < 1 in OS was found for FOXP4 (EC), whereas for HR > 1 DYM, PDAP1, and CHP1 were identified for EC, MC, and CCC, respectively (Fig. 5a). For DSS, HR > 1 was estimated in both stage groups for HBG1 (HGSC) and ASPM (CCC), and HR < 1 for DCPS (EC; Fig. 5b). Inversely, the risk association differed between the stage groups for some proteins. For OS, BLNK (HGSC) and CHML (CCC) displayed a decreased risk of death at early-stage but increased risk of death at late-stage. CCT4 (HGSC) was associated with favorable prognosis at early stage and unfavorable prognosis at late stage (Fig. 6a). For DSS, CCT5 (HGSC) and RPL31 (CCC) had HR < 1 at late-stage and HR > 1 at early-stage, and CHML had HR > 1 at late-stage and HR < 1 at early-stage which is in line with the estimations in OS for CHML (Fig. 6b).

### Involvement of prognostic proteins in biological processes

**Fig. 7:**
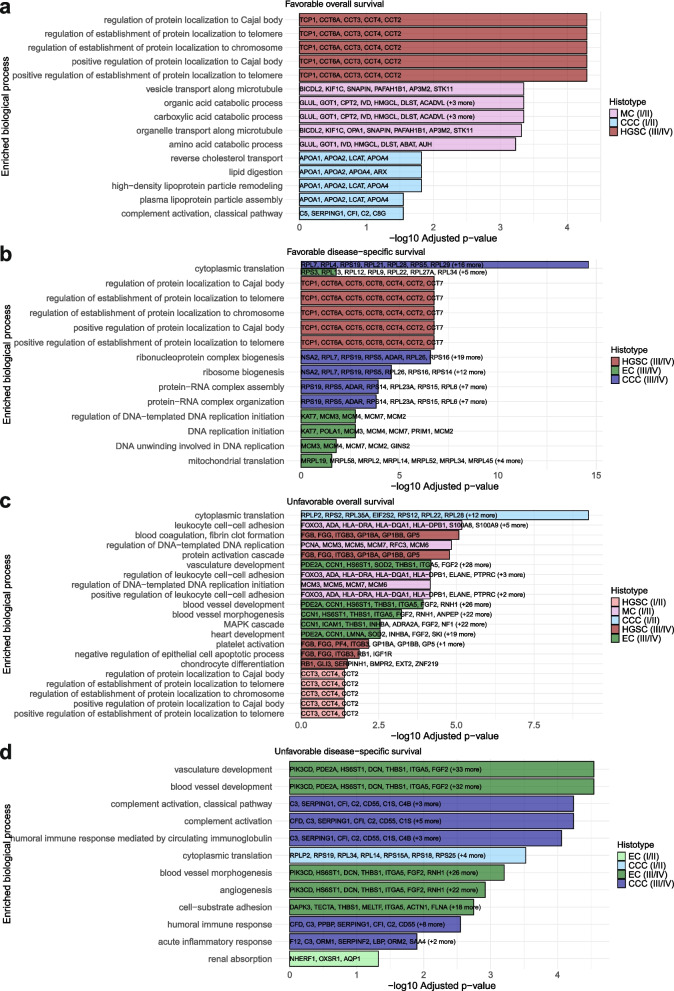
Gene ontology enrichment analysis of proteins significantly associated with decreased and increased risk of death. The five most significantly enriched (adjusted p-value < 0.05) biological processes along with involved proteins at early- and late-stage disease for (**a**) favorable prognosis for OS, (**b**) favorable prognosis for DSS, (**c**) unfavorable prognosis for OS, and (**d**) unfavorable prognosis for DSS. Adjusted p-value were corrected using the Benjamini–Hochberg model. CCC Clear cell ovarian carcinoma, DSS Disease-specific survival, EC Endometrioid ovarian carcinoma, HGSC High-grade serous ovarian carcinoma, MC Mucinous ovarian carcinoma, OS Overall survival

For proteins found to be significantly associated with clinical outcome, GO enrichment analysis (GOEA) was performed to identify biological processes (BPs) linked to favorable or unfavorable prognosis across histotypes at early and advanced disease stage, along with the proteins involved in these processes. For decreased risk of death (HR < 1) in OS, significantly enriched (adjusted p-value < 0.05) BPs were found for MC and CCC at early stage and HGSC at late stage. BPs for MC were primarily linked to catabolic and microtubule processes (GLUL and GOT1 were shared proteins across all processes), whereas reverse cholesterol transport was the most enriched pathway for CCC while lipoprotein formation processes were also highly enriched. Proteins from the APOA-family were involved in all BPs except complement activation for CCC. For HGSCs at late stage, all significantly enriched BPs were connected to protein localization of cajal body, telomere, and chromosome with TCP1 and CCT-proteins driving this enrichment (Fig. 7a). For DSS, no significantly enriched BPs were found at early stage, but significant BPs was found for HGSC (n = 52), EC (n = 8), and CCC (n = 14) at late stage. Dysregulation of protein localization of cajal body, telomere and chromosomes were, as for OS for HGSC, enriched in DSS as well for HGSC. The pathways for HGSC aligned with the observations at late stage for OS with the same proteins involved. Initiation and regulation of DNA replication appeared to be a hallmark of EC (KAT7 and MCM-family proteins) as well as cytoplasmic translation which was also shared with CCC (multiple RPL- and RPS-proteins involved in the BPs for both histotypes). The most enriched BPs for CCC at late stage for DSS were, apart from cytoplasmic translation, biogenesis of ribosomal constituents as well as protein-RNA complex assembly and organization. RPL- and RPS proteins were shared among these processes, but NSA2 was uniquely involved in biogenesis (Fig. 7b; Data S8).

For increased risk of death (HR > 1) for OS, BPs associated with decreased risk of death for HGSC at late stage were instead associated with increased risk of death for early-stage patients for this histotype, with overlapping CCT-proteins involved in both stage groups. This was also true for cytoplasmic translation in CCC. This process was associated with favorable outcome at late stage, but unfavorable outcome at early stage for OS. Moreover, leukocyte cell–cell adhesion (FOX03, ADA, and HLA-proteins common) and regulation of DNA-templated DNA initiation and replication (MCM-proteins) were the most enriched processes for MC at early stage for OS. At late stage on the other hand, increased risk of death appeared to be connected to blood clot formation, platelet activation, protein activation cascade, and negative epithelial cell apoptosis for HGSC with FGB and FGG involved in all of them. For EC, poor prognosis at late stage was mainly driven by processes related to blood vessel development, vasculature development, and the MAPK cascade with the proteins PDE2A and CCN1 frequently being involved in all processes (Fig. 7c). In DSS, enriched BPs associated with unfavorable outcomes were found for only EC and CCC at early stage. Again, cytoplasmic translation was the most enriched process (here the only process) for CCC which matches the findings for OS. For EC, renal absorption was the only enriched BP, driven by NHERF1, OXSR1, and AQP1. In DSS for late-stage samples, the significantly enriched pathways for HGSC were related to immune response, with SERPING1 and C-proteins involved in the processes. For EC, the findings greatly overlapped with the equivalent analysis for late stage in OS, with the involvement of similar proteins (Fig. 7d; Data S8, S9).

## Discussion

Ovarian cancer is the most lethal gynecologic malignancy, but the available clinical tools are limited and have suboptimal performance [19, 20]. Currently, there are no available tools with histotype-specificity for early- or late-stage disease. Therefore, we need novel biomarkers that can molecularly distinguish the histotypes at different stages, identify patient risk groups, and provide therapeutic targets. Such biomarkers could provide novel detection tools and guide treatment decisions based on histotype and stage, which can aid in advancing screenings and patient care. Consequently, we performed a comprehensive proteomics analysis of 244 EOCs stratified by histotype and stage. Differential abundance analysis revealed proteins with no stage-specificity (S100A1, SNCG, CRABP2, AGR2, CTH), early stage-specificity (TSPYL5, VWA2, SCGB1D2, PIGR2) and late stage-specificity (IL4I1, GPC6, S100P). Biomarker panels to stratify histotypes highlighted S100A1 as a key protein to distinguish HGSC from other histotypes. Survival analysis identified histotype- and stage-specific prognostic proteins associated with favorable (EXO3CL2, PPIL6, VPS35L, SHKBP1) and unfavorable (GYG1, MRPS24, GAPDH) outcomes. Enrichment of BPs identified cytoplasmic translation to be a hallmark for increased risk of death for CCC, whereas localization of cajal body, telomere, and chromosomes appeared to differ in its association with prognosis between early- and late stage HGSC.

Profiling the abundance of proteins may provide proteins detectable in a histotype at a certain stage of the disease at an upregulated level compared to the other histotypes or alternatively be absent, and may serve as complementary tools for diagnosis, histopathology, staging, and therapeutic targets. We identified DAPs by performing differential abundance analysis. The number of DAPs for each histotype suggested that EC has similar protein abundances compared to the other histotypes, indicated by the low number of DAPs in early stage and none in late stage. This finding has been verified previously on the gene expression-level using microarrays [21]. S100A1, SNCG, and CRABP2 emerged as highly upregulated in HGSC in both early and advanced stage. S100A1 is known to be overexpressed in EOC compared to healthy tissue, but has also shown potential to inhibit cell proliferation [22, 23]. Moreover, SNCG promotes metastasis and CRABP2 chemoresistance in EOC [24, 25]. Across all stages, AGR2 and CTH were the most upregulated proteins in MC and CCC, respectively. The mRNA expression of AGR2 in early-stage MC has been observed in previous studies, where its overexpression was marked by cell proliferation and migration as well as serum secretion [26]. A proteomic study performed by Cochrane et al. confirmed the CCC-specificity of CTH upregulation [27]. Additionally, ARID3A has been shown to promote stem cell generation, which may contribute to chemoresistant traits observed in late stages [28]. This warrants further investigation into the role of ARID3A in CCC.

The upregulation of TSPYL5 (HGSC), VWA2 (EC), SCGB1D2 (MC), and PIGR (CCC) was specific for early-stage patients, with VWA2 also being upregulated in early stage compared to late stage within EC. Cell migration and evasion is evidenced to be blocked by TSPYL5 in EOC, while the role of VWA2 in EOC is not known but is suggested as a prognostic biomarker in colorectal cancer [29, 30]. EOC overexpression of SCGB1D2 has been shown to promote cell proliferation, whereas downregulation is associated with favorable prognosis in HGSC [31, 32]. In late stage, the histotypes were characterized by upregulation of IL4I1 (HGSC), GPC6 (MC), and VNN1 (CCC). High levels of the amino acid-metabolizing IL4I1 protein have been observed in advanced HGSC, and its downregulation can inhibit proliferation, migration, and invasion of cells in EOC [33, 34]. Less is known about GPC6, but it has been shown to be a favorable prognostic marker for early-stage EOC by predicting CD8 + T-lymphocyte infiltration [35]. Lastly, VNN1 is a proposed biomarker for early detection of pancreatic cancer and prognostic biomarker for colorectal cancer [36, 37]. It is important to note that these findings have not yet been validated with external datasets or immunohistochemistry. Such validation represents an essential next step for assessing the potential clinical utility of the identified proteins in EOC.

The SVM modeling utilized DAPs to identify protein panels distinguishing the histotypes from each other in early- and late stage based on relative abundance. Some proteins with the highest and lowest log2 FC were chosen for inclusion in the panels such as SNCG for HGSC (early-stage), CTH (early-stage), ARID3A (late-stage), and ANXA4 (both stage groups) for CCC, and CEACAM5 (early-stage) and LGALS4 (both stage groups) for MC. In addition, SLC7A5 was specific for the late-stage CCC panel, and has previously been shown to enhance Olaparib-resistance in EOC [38]. As CDH6 and SPON1, which were early-stage specific for HGSC, have been identified in plasma and serum of EOC patients, additional validation of their diagnostic implications should be assessed [39, 40]. Notably, the protein HIP1R which was downregulated in HGSC, was included in the biomarker panel with the highest AUC score for both stage groups for HGSC. HIP1R has previously been reliably identified in ovarian cancer ascites, and has also been associated with improved prognosis in early-stage lung adenocarcinoma, where it appears to suppress metastasis [41, 42]. Biomarker panels from pairwise comparisons suggested that S100A1 appears to be a key protein for distinguishing HGSC from other histotypes in late-stage. While multiple DAPs were able to collectively separate HGSC from EC in early-stage, only CRACR2B was selected for the model for the late-stage comparison. These combinations of dysregulated proteins warrant further validation with external cohorts to assess their predictive potential and may help define panels with translational utility, especially considering the low sample numbers for early-stage MC and CCC, which were shown to impact the SVM modeling.

Multivariate Cox regression with bootstrap validation was performed to investigate the prognostic potential of all identified proteins. For OS and DSS, we were able to identify prognostic proteins for the histotypes in both stage groups. High abundance of PPIL6 was associated with favorable prognosis for HGSC in early-stage, of which its involvement in cancer is not known. The favorable protein EXO3CL2 (EC in late stage) on the other hand is evidenced to promote angiogenesis in mice [43]. For unfavorable prognosis, GYG1 (EC in late stage) has previously been suggested as a therapeutic target owing to its role in glycogen metabolism, and in this survival analysis was specific for EC in late-stage for both OS and DSS [44]. Both MRPS24 (HGSC in early stage) and SNCG have previously been linked to poor survival, but notably poor OS based on SNCG was found for MC in early-stage here which does not correlate with its HGSC-specificity in previous studies [24, 45]. Among the prognostic proteins for DSS, high abundance of VPS35L (HGSC in late stage) and SHKBP1 (HGSC in early stage) was associated with favorable outcomes, both having been suggested as biomarkers of diagnostic and therapeutic potential in other cancer types, respectively [46, 47]. The poor prognosis for patients with high abundance of GAPDH (HGSC in early-stage) suggests that this protein, that previously has showed unfavorable outcomes in advanced HGSC, may also indicate increased risk for early-stage patients [48].

Proteins positively correlated with clinical outcomes in early- and late stage are of value as their role as biomarkers would be stage-independent. Here, FOXP4 (EC) was revealed as a favorable protein while DYM (EC), PDAP1 (MC), and CHP1 (CCC) were unfavorable in OS. While conflicting evidence for the prognostic power of FOXP4 in EOC has been demonstrated in previous studies and the role of DYM is largely unknown, PDAP1 and CHP1 have been identified as carcinogenesis and cell proliferation-inducing components in other cancers [49–52]. Equivalent proteins for DSS have also shown prognostic value, such as the favorable protein DCPS for EC [53]. Notably, the identification of ASPM as an unfavorable protein for CCC is a proposed prognostic biomarker in EOC, also showing low levels in serous tissue [54]. On the other hand, BLNK (HGSC for OS) and CHML (CCC for OS and DSS) displayed a decreased risk of death at early-stage and increased risk of death at late-stage. Inversely, favorable prognosis at late-stage and unfavorable at early-stage was observed for CCT4 (HGSC for OS), CCT5 (HGSC for DSS), and RPL31 (CCC for DSS). These findings warrant further investigation into the underlying pathways these proteins are involved in to gain a better understanding of the mechanisms leading to differences in outcome between early and advanced disease and whether these biomarkers could be used as prognostic and/or therapeutic targets.

GO enrichment analysis of the identified prognostic proteins showed enriched BPs that may be key prognostic factors at different disease stages in each histotype. Favorable OS at early stage was marked by catabolic processes in MC, and reverse cholesterol transport and lipoprotein formation in CCC. Catabolism affects chemosensitivity and cholesterol transportation affects growth and migration of cells in EOC [55, 56]. The localization of cajal body, telomeres, and chromosomes in HGSC was the only enriched BPs for favorable outcome at late stage for OS and DSS. Since poly-(ADP-ribose) polymerase 1 is critical for protein localization to cajal body, this process may be involved in the sensitivity to PARP inhibitors [57]. Further investigation is required to determine whether processes related to Cajal body dynamics have mechanistic relationship to PARP inhibitor sensitivity. At present, these association are hypothetical and should not be interpreted as evidence for involvement in chemosensitivity. Favorable DSS indicated that initiation and regulation of DNA replication, a known stressor in cancer, is a key pathway in EC at late stage where the MCM-proteins were involved, which aligns with previous research [58, 59]. Cytoplasmic translation, which was shared between EC and CCC but more enriched in CCC for favorable DSS at late stage, is a process of which its specific role in cancer is not understood yet.

Interestingly, BPs involved in unfavorable OS indicated that localization of cajal body, telomeres, and chromosomes as well as cytoplasmic translation, which were associated with favorable prognosis at late stage for HGSC and CCC respectively, were instead BPs connected to unfavorable prognosis at early stage for the two histotypes with similar proteins involved. These processes could therefore be of particular interest when studying the survival of HGSC and CCC patients. Moreover, enrichment of initiation and regulation of DNA replication, which was enriched in favorable prognosis for EC at late stage, was also one of the most significantly enriched BPs for favorable prognosis for MC in early stage, although regulation of leukocyte cell–cell adhesion also appears to be a key factor in MC. Leukocyte cell adhesion molecules have emerged as prognostic candidates for aggressive type II EOC tumors [60]. Poor prognosis at late stage highlighted BPs related to angiogenesis, a well-studied event used for potential novel targeted therapies, to be a hallmark of EC in both OS and DSS [61]. Blood clotting, which indicated poor OS in late-stage HGSC, has formerly been identified for advanced EOC, but not for HGSC in particular [62]. Lastly, increased risk of death for CCC patients at late stage may be caused by immune response-related processes as identified here in DSS. Overexpression of interleukins has been shown to promote resistance to immunotherapy in EOC, so these BPs may contribute to treatment resistance in advanced CCC [63].

Although the patient cohort is large, some sample groups are small since the cohort is subdivided into histotypes, and within histotypes into early- and late stage. This is especially true for MC and CCC, where SVM models suffered from overfitting. Survival analysis may have suffered over- and underestimation of hazard ratios, inflated confidence intervals, and high bootstrap p-values due to model instability as a result, as indicated by moderate C-indexes. Future studies could greatly benefit from larger cohorts for EC, MC, and CCC, especially for early-stage samples. The findings should be subjected to external validation using publicly available data as well as immunohistochemistry or western blot analyses to assess discriminatory power. Additionally, elucidating the roles of the enriched biological processes in tumorigenesis and chemoresistance will require targeted functional studies, including drug response assays. While available proteomics data for EOC exists, they are scarce and often not stratified by histotype. For many of these datasets, it is not clear if reclassification according to the WHO 2020 guidelines has been performed and survival data are seldom provided. Therefore, external validation was not performed in the present study.

## Conclusions

This study identified distinct deregulated histotype-specific proteins, with both stage-specific and non-stage-specific proteins. Biomarker panels comprising DAPs were able to distinguish between the histotypes with high predictive power, thereby providing novel tools for diagnostic purposes and could be complementary to conventional histopathology and FIGO staging. Survival analysis found stage-specific proteins strongly associated with clinical outcomes for each histotype. GO enrichment of these proteins enabled mapping of dysregulated biological processes connected to survival and the proteins involved. This enables identification of disease-driving mechanisms as well as traits resulting in more favorable outcomes, while the involved proteins suggest key players in these processes that can be subjected to further targeted studies. While these proteins could hold prognostic value or represent future therapeutic targets, such implications remain uncertain at this stage. It is important to note that this is an explorative study and additional work incorporating external cohorts, protein-level validation and evaluation in less invasive sample types, will be needed to assess their diagnostic and prognostic potential.

## Supplementary Information

Supplementary Material 1.

Supplementary Material 2.

## Data Availability

The mass spectrometry and Spectronaut analysis files have been deposited to the MassIVE repository with the dataset identifier MSV000097588 or using the link https://massive.ucsd.edu/ProteoSAFe/dataset.jsp?task=d49017f42a1e482299a6d9d0f9557b15. Metadata and protein quantities have also been uploaded to Zenodo at 10.5281/zenodo.17856568. All analysis code has been uploaded and is publicly available at https://github.com/lwernerGU/Proteomics-analysis-pipeline, named ESvsLSproteomics.R.

## Abbreviations

EOC: Epithelial ovarian cancer
HGSC: High-grade serous ovarian carcinoma
LGSC: Low-grade serous ovarian carcinoma
EC: Endometrioid ovarian carcinoma
MC: Mucinous ovarian carcinoma
CCC: Clear-cell ovarian carcinoma
DAP: Differentially abundant protein
SVM: Support vector machine
LASSO: Least absolute shrinkage and selection operator
AUC: Area under the curve
FC: Fold change
FDR: False discovery rate
OS: Overall survival
DSS: Disease-specific survival
HR: Hazard ratio
BP: Biological process
GO: Gene ontology
GOEA: Gene ontology enrichment analysis
