# Supplementary material for: Proteomic analysis of ovarian carcinoma reveals diagnostic and prognostic biomarkers with histotype- and stage-specificity
Source: J Ovarian Res. 2026 Jan 28;19:74. doi: 10.1186/s13048-026-01984-4 (PMC12924536; doi:10.1186/s13048-026-01984-4)
Supplement: Supplementary file 2 — Supplementary Material 2. [file 13048_2026_1984_MOESM2_ESM.pdf]

Supplementary information (SI)

Figures and tables

Article title

Proteomic analysis of ovarian carcinoma reveals diagnostic and prognostic biomarkers with histotype- and stage-specificity

Journal

Journal of Ovarian Research

Authors

Lucas Werner, Ella Ittner, Hugo Swenson, Elisabeth Werner Rönnerman, Claudia Mateoiu, Anikó Kovács, Pernilla Dahm-Kähler, Per Karlsson, Toshima Z. Parris, Khalil Helou

Corresponding author

Lucas Werner, lucas.werner@gu.se

Supplementary figures

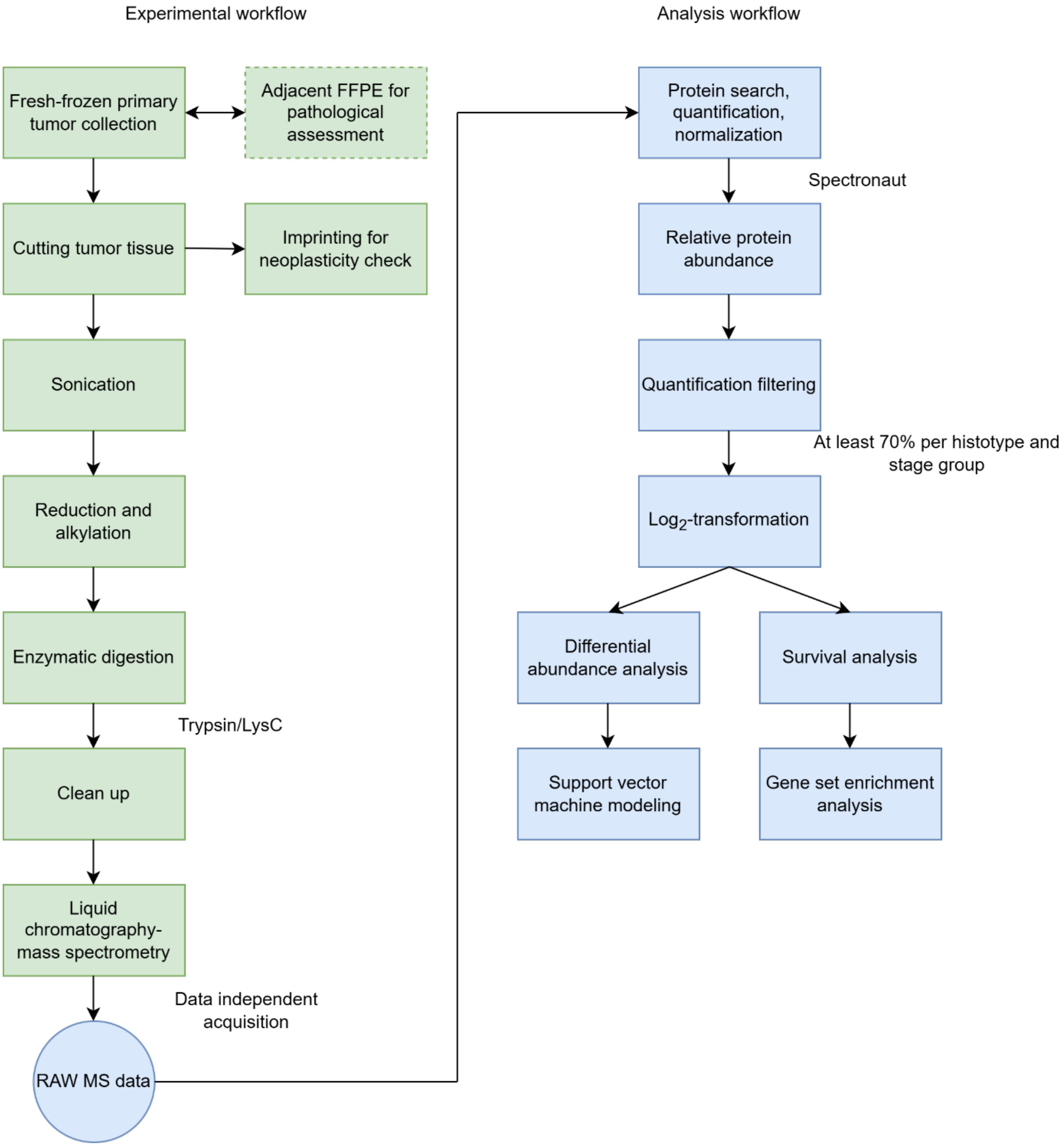

Fig. S1

Schematic overview of the experimental (green) and analytical (blue) workflow of the study.

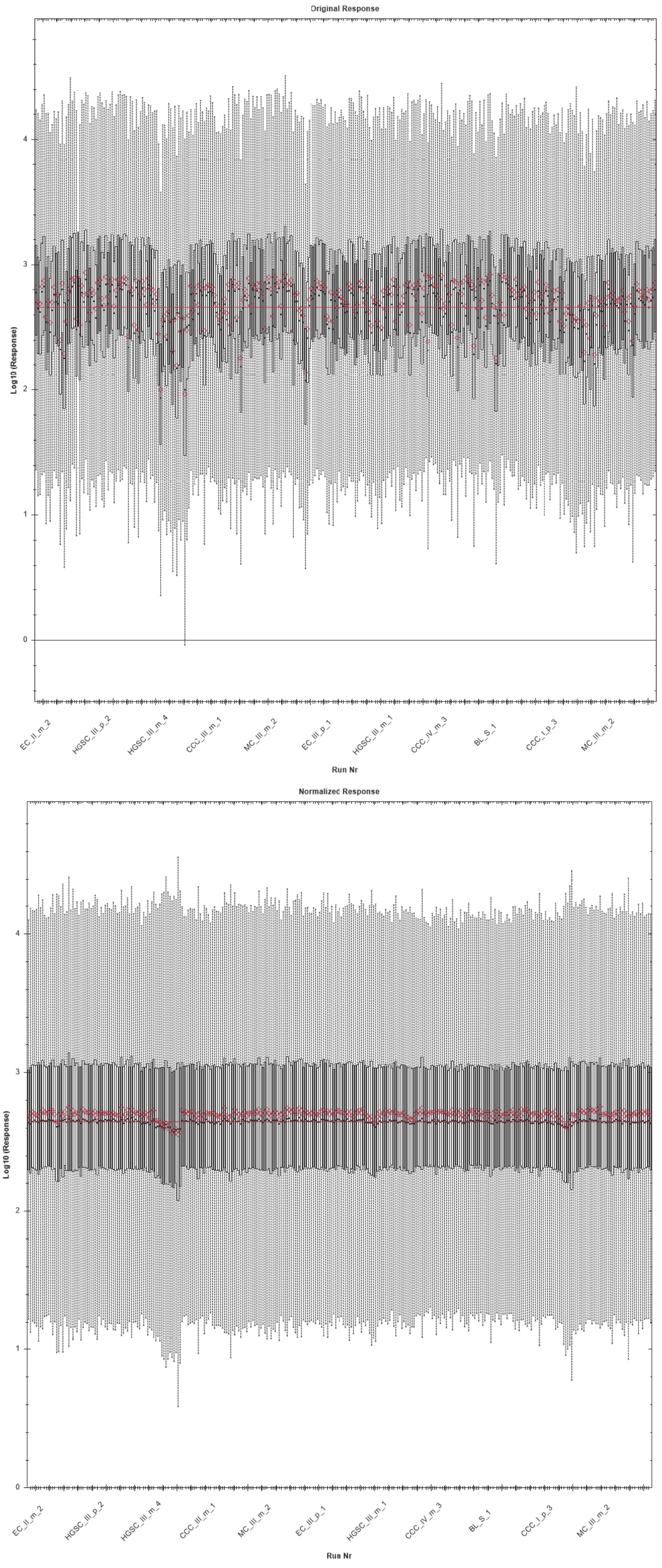

Fig. S2

Boxplots over the intensity distribution of all samples before (top) and after (bottom) cross-run normalization in Spectronaut.

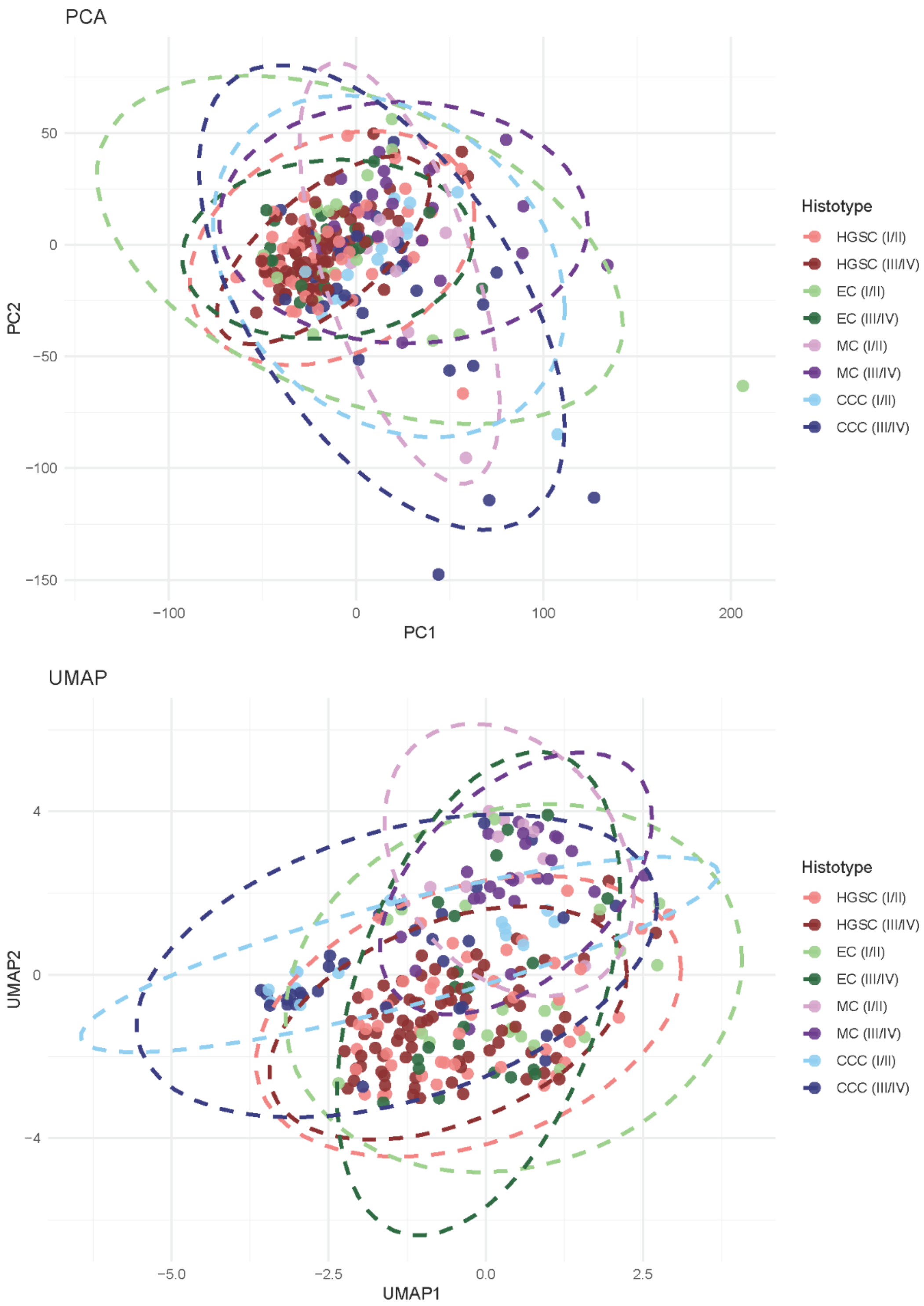

Fig. S3

Sample intensity distribution. Displayed is the intensity reduced to the two main principal components in PCA (top) and UMAP (bottom) of data stratified by histotypes in early-stage (I/II) and late-stage (III/IV) groups after normalization, log2-transformation, and filtering for at least 70% quantification in at least one histotype stage group. *CCC* Clear-cell ovarian carcinoma, *EC* Endometrioid ovarian carcinoma, *HGSC* High-grade serous ovarian carcinoma, *MC* Mucinous ovarian carcinoma, PCA Principal component analysis, UMAP Uniform manifold approximation and projection.

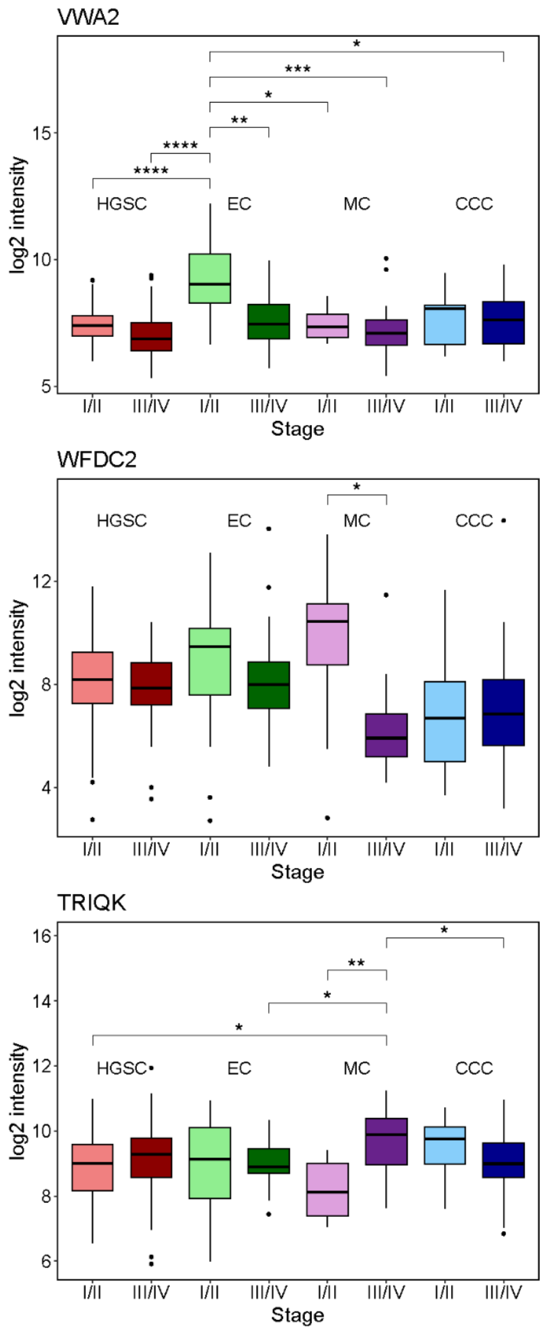

Fig. S4

Histograms of proteins differentially abundant between early- and late stages within a histotype. Displayed are the identified proteins of highest log2 FC when comparing abundances in early stages (I or II) to late stages (III or IV) within a histotype for the histotypes where such proteins were identified after first filtering for statistical significance (FDR < 0.05). The *p*-values were derived from pairwise Wilcoxon tests and FDR corrected. The middle horizontal lines represent the median of the log2 intensity, upper and lower bounds the 25th and 75th percentiles, whiskers the largest intensities 1.5 times outside the percentiles and dots outliers outside these ranges. CCC Clear cell ovarian carcinoma, DAPs Differentially abundant proteins, EC Endometrioid ovarian carcinoma, FC Fold change, FDR False discovery rate, HGSC High-grade serous ovarian carcinoma, MC Mucinous ovarian carcinoma, * FDR < 0.05, ** FDR ≤ 0.01, *** FDR ≤ 0.001, **** FDR ≤ 0.0001.

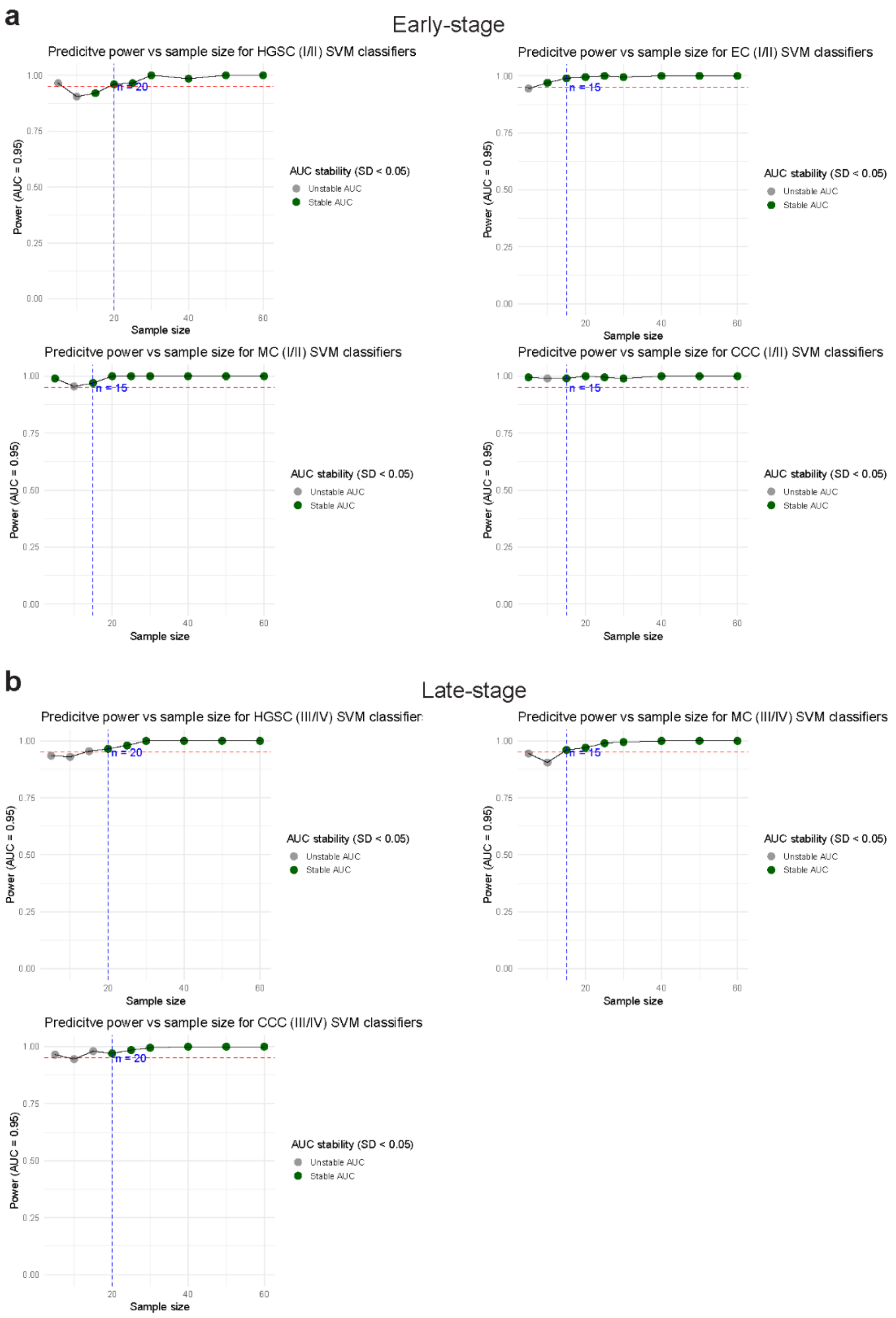

Fig. S5

Curves over the simulated AUC score with increasing sample size for biomarker panels for each histotype. The plot depicts the simulated AUC score based on the intensity data of selected biomarkers from the SVM models for histotypes in early stages in **a)**, and late stages in **b)**. Simulations were supplied with synthetic (duplicated data) when sample size exceeded the actual number of samples in the cohort. Indicated in blue is the theoretical number of samples for AUC ≥ 0.95 (red dashed line), when the sample size is at least greater than 10, and the AUC score has a standard deviation < 0.05 (green). AUC Area under the curve, *CCC* Clear-cell ovarian carcinoma, *EC* Endometrioid ovarian carcinoma, *HGSC* High-grade serous ovarian carcinoma, *MC* Mucinous ovarian carcinoma, SVM Support vector machine.

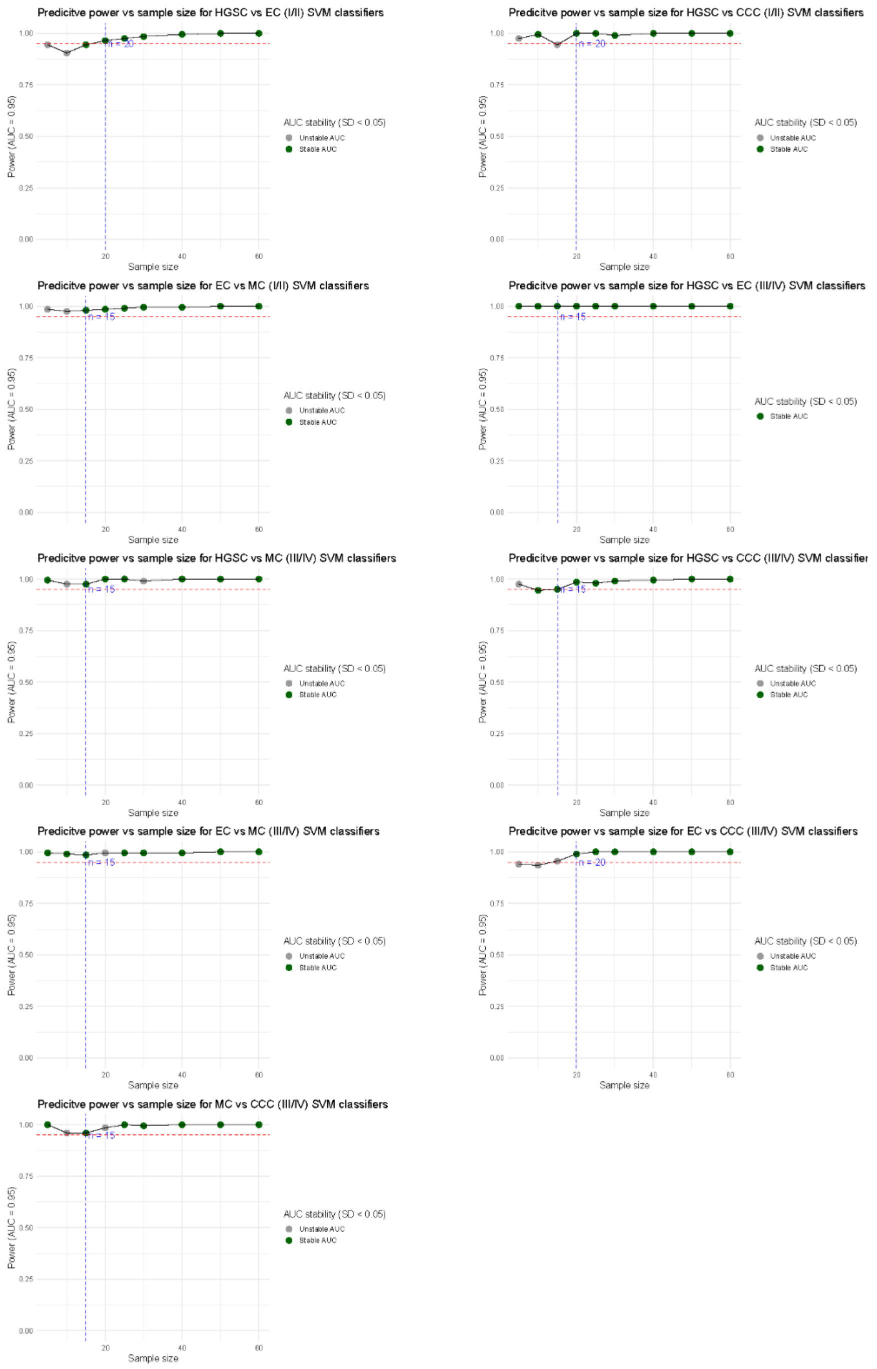

Fig. S6

Curves over the simulated AUC score with increasing sample size for biomarker panels for each pairwise comparisons of histotypes in early- and late stages. The plot depicts the simulated AUC score based on the intensity data of selected biomarkers from the SVM models. Simulations were supplied with synthetic (duplicated data) when sample size exceeded the actual number of samples in the cohort. Indicated in blue is the theoretical number of samples for AUC ≥ 0.95 (red dashed line), when the sample size is at least greater than 10, and the AUC score has a standard deviation < 0.05 (green). AUC Area under the curve, *CCC* Clear-cell ovarian carcinoma, *EC* Endometrioid ovarian carcinoma, *HGSC* High-grade serous ovarian carcinoma, *MC* Mucinous ovarian carcinoma, SVM Support vector machine.

Supplementary tables

Table S1

Most differentially abundant proteins at early-stage and late-stage for each histotype.

**Upregulated**					
**Histotype**	**Stage group**	**Protein accession**	**Protein symbol**	**FDR**	**Log2 FC**
HGSC	I, II	P23297	S100A1	8.23e-6	2.27
	I, II	O76070	SNCG	2.80e-7	2.17
	I, II	P29373	CRABP2	9.30e-7	1.85
	I, II	P42771	CDKN2A	4.19e-5	1.48
	I, II	P23219	PTGS1	2.65e-5	1.41
	III, IV	P23297	S100A1	3.92e-12	2.67
	III, IV	O76070	SNCG	3.42e-13	2.36
	III, IV	P29373	CRABP2	3.92e-12	1.99
	III, IV	Q9HCB6	SPON1	1.05e-7	1.63
	III, IV	P23219	PTGS1	3.11e-8	1.39
EC	I, II	Q5GFL6	VWA2	4.10e-6	1.82
	I, II	Q9ULR3	PPM1H	1.62e-3	1.08
	I, II	Q8J025	APCDD1	2.89e-2	1.00
	I, II	Q9NQ66	PLCB1	3.87e-2	0.65
	I, II	P51648	ALDH3A2	7.64e-3	0.65
	III, IV	-	-	-	-
MC	I, II	O95994	AGR2	1.94e-6	4.07
	I, II	P01833	PIGR	1.10e-6	4.04
	I, II	P56470	LGALS4	1.58e-5	3.78
	I, II	Q6UX06	OLFM4	1.22e-5	3.76
	I, II	Q9Y6R7	FCGBP	4.42e-8	3.43
	III, IV	O95994	AGR2	1.25e-14	4.22
	III, IV	P06731	CEACAM5	3.46e-5	2.82
	III, IV	P14091	CTSE	1.59e-6	2.82
	III, IV	P19075	TSPAN8	2.26e-12	2.81
	III, IV	Q9Y6R7	FCGBP	2.66e-12	2.74
CCC	I, II	P32929	CTH	1.60e-19	3.09
	I, II	Q8IZV5	RDH10	1.64e-10	2.44
	I, II	P19801	AOC1	1.04e-6	2.27
	I, II	Q9Y617	PSAT1	1.81e-6	2.22
	I, II	P09525	ANXA4	1.98e-11	2.21
	III, IV	P32929	CTH	2.59e-19	2.15
	III, IV	O95497	VNN1	2.92e-9	2.10
	III, IV	Q6ZVX7	NCCRP1	3.29e-14	2.09
	III, IV	O43272	PRODH	9.70e-11	2.09
	III, IV	Q9Y2T3	GDA	1.02e-8	2.06
**Downregulated**					
HGSC	I, II	P01833	PIGR	6.56e-11	-3.13
	I, II	O95994	AGR2	3.46e-8	-3.02
	I, II	Q9Y2T3	GDA	7.78e-8	-2.02
	I, II	O60635	TSPAN1	4.98e-5	-1.96
	I, II	Q93099	HGD	4.58e-5	-1.85
	III, IV	O95994	AGR2	8.00e-11	-2.67

	III, IV	P14091	CTSE	6.80e-6	-2.63
	III, IV	O60635	TSPAN1	2.17e-9	-2.17
	III, IV	P01833	PIGR	7.53e-9	-2.08
	III, IV	P25815	S100P	5.41e-9	-2.02
EC	I, II	Q13421	MSLN	1.62e-3	-2.12
	I, II	P00966	ASS1	1.62e-3	-1.44
	I, II	Q96DC8	ECHDC3	4.20e-2	-1.18
	III, IV	-	-	-	-
MC	I, II	P23297	S100A1	3.27e-4	-3.28
	I, II	Q9BQI0	AIF1L	1.03e-4	-1.90
	I, II	P42771	CDKN2A	6.20e-3	-1.88
	I, II	P50895	BCAM	4.73e-4	-1.85
	I, II	Q9Y617	PSAT1	3.51e-3	-1.66
	III, IV	P23297	S100A1	6.66e-8	-3.04
	III, IV	Q7Z7D3	VTCN1	1.50e-4	-2.38
	III, IV	P50895	BCAM	2.11e-10	-2.08
	III, IV	P42771	CDKN2A	7.72e-7	-1.91
	III, IV	O95436	SLC34A2	2.46e-4	-1.80
CCC	I, II	P29373	CRABP2	2.38e-3	-1.83
	I, II	P40424	PBX1	3.81e-6	-1.71
	I, II	Q14508	WFDC2	3.78e-2	-1.70
	I, II	Q9HCY8	S100A14	4.68e-2	-1.66
	I, II	Q96SQ9	CYP2S1	1.64e-3	-1.66
	III, IV	O76070	SNCG	5.35e-6	-1.99
	III, IV	P29373	CRABP2	4.22e-7	-1.89
	III, IV	Q9HCB6	SPON1	1.25e-5	-1.75
	III, IV	P23297	S100A1	7.14e-3	-1.47
	III, IV	P40424	PBX1	3.63e-8	-1.33

The five proteins of highest log2 fold changes for up- and downregulation for each histotype in early-stage and late-stage. *CCC* Clear-cell ovarian carcinoma, *EC* Endometrioid ovarian carcinoma, *FDR* False discovery rate, *HGSC* High-grade serous ovarian carcinoma, *MC* Mucinous ovarian carcinoma, *log 2 FC* log2 fold change. Displayed log2 FC values for the protein for a histotype when compared to the abundance of the other histotypes.

Table S2

Most differentially abundant and unique proteins at early-stage and late-stage for each histotype.

**Upregulated**					
**Histotype**	**Stage group**	**Protein accession**	**Protein symbol**	**FDR**	**Log2 FC**
HGSC	I, II	Q86VY4	TSPYL5	3.36e-7	1.33
	III, IV	Q96RQ9	IL4I1	8.67e-6	1.38
EC	I, II	Q5GFL6	VWA2	4.10e-6	1.82
	III, IV	-	-	-	-
MC	I, II	O95969	SCGB1D2	3.05e-3	2.90
	III, IV	Q9Y625	GPC6	1.20e-7	1.91
CCC	I, II	P01833	PIGR	4.18e-2	1.78
	III, IV	O95497	VNN1	2.92e-9	2.10
**Downregulated**					
HGSC	I, II	P08263	GSTA1	0.02	-1.52
	III, IV	P14091	CTSE	6.80e-6	-2.63

EC	I, II	Q13421	MSLN	1.62e-3	-2.12
	III, IV	-	-	-	-
MC	I, II	Q9HCB6	SPON1	4.64e-2	-1.54
	III, IV	Q7Z7D3	VTCN1	1.50e-4	-2.38
CCC	I, II	Q14508	WFDC2	3.78e-2	-1.70
	III, IV	O76070	SNCG	5.35e-6	-1.99

The proteins of highest log2 fold changes for up- and downregulation for each histotype in early-stage and late-stage after filtering for proteins that are shared between early-stage and late-stage within each histotype. *CCC* Clear-cell ovarian carcinoma, *EC* Endometrioid ovarian carcinoma, *FDR* False discovery rate, *HGSC* High-grade serous ovarian carcinoma, *MC* Mucinous ovarian carcinoma, *log 2 FC* log2 fold change. Displayed log2 FC values for the protein for a histotype when compared to the abundance of the other histotypes.

Table S3

Summary statistics for significant multivariate Cox regression.

**Overall survival**			
**Histotype**	**Stage**	**HR < 1**	**HR > 1**
HGSC	I, II	38	105
	III, IV	132	110
EC	I, II	50	54
	III, IV	158	277
MC	I, II	70	51
	III, IV	30	88
CCC	I, II	127	154
	III, IV	61	200
**Disease-specific survival**			
HGSC	I, II	31	146
	III, IV	208	87
EC	I, II	18	35
	III, IV	216	340
MC	I, II	0	0
	III, IV	98	135
CCC	I, II	73	106
	III, IV	144	294

Number of proteins significantly (FDR < 0.05, bootstrap *p*-value < 0.20) associated increased risk (HR > 1) and decreased risk (HR < 1) of death in OS and DSS at early-stage and late-stage for each histotype. HRs were estimated from multivariate Cox regression adjusted for age and stage of the patients. *CCC* Clear cell ovarian carcinoma, *DSS* Disease-specific survival, *EC* Endometrioid ovarian carcinoma, *FDR* False discovery rate, *HGSC* High-grade serous ovarian carcinoma, *HR* Hazard ratio, *MC* Mucinous ovarian carcinoma.
